# Supplementary material for: A Likelihood Approach for Real-Time Calibration of Stochastic Compartmental Epidemic Models
Source: PLoS Comput Biol. 2017 Jan 17;13(1):e1005257. doi: 10.1371/journal.pcbi.1005257 (PMC5240920; doi:10.1371/journal.pcbi.1005257)
Supplement: S1 File — (TAR.GZ) [file pcbi.1005257.s014.tar.gz › HSPH_Online-SI-Revision/EnKF07/n1-mild/n1-mild_EnKF_table4.pdf]

1	R0	{1.27677, 7.89375}	{2.71933, 6.14957}	{{2.45086, 0.208146}, {3., 9.17652}}	{113.782, 38.9007}	inf	18.	77.8673	{11.0739, 163.224}	351.645	0.418657	newly	14.	71.93	{7., 211.}	434.643	0.482883	Reff	0.891185	0.479548	{0.104845, 0.715904}	65.7934	ar	400.	1743.92	{1546., 2415.}	335.98
2	R0	{1.33231, 10.5259}	{2.84252, 4.80081}	{{2.54389, 1.71776}, {3., 6.67418}}	{113.353, 54.3905}	inf	9.	40.7166	{3.80132, 60.3764}	379.857	0.498271	newly	6.	59.25	{4., 120.}	907.167	0.645955	Reff	0.735435	0.541506	{0.0267477, 1.10746}	64.6885	ar	467.	1642.76	{1493., 2053.}	251.769
3	R0	{1.2273, 6.76944}	{2.47621, 6.06566}	{{1.78774, 1.66556}, {3., 8.0469}}	{109.256, 26.4798}	inf	10.	42.3218	{5.11452, 56.0705}	343.876	0.490177	newly	13.	48.61	{4., 99.}	309.154	0.457533	Reff	0.924157	0.468947	{0.0726796, 0.836305}	59.5478	ar	314.	1730.36	{1579., 1979.}	451.07
4	R0	{1.41215, 17.5742}	{2.70392, 9.02009}	{{2.24256, 4.51933}, {3., 11.2283}}	{93.3785, 48.9094}	inf	22.	57.882	{15.8471, 104.814}	178.1	0.407263	newly	12.	42.83	{7., 69.}	272.25	0.511859	Reff	1.14808	0.52202	{0.113874, 1.15504}	68.1118	ar	465.	1806.57	{1630., 2209.}	288.51
5	R0	{1.42222, 6.15886}	{2.48866, 6.64665}	{{1.31853, 3.33928}, {3., 11.7557}}	{78.5967, 46.6483}	inf	19.	47.9853	{15.928, 66.5543}	161.722	0.346956	newly	13.	59.86	{11., 84.}	368.615	0.441039	Reff	0.797865	0.548886	{0.130618, 1.23218}	71.013	ar	490.	1744.13	{1577., 2252.}	255.945
6	R0	{1.24363, 9.95596}	{2.65843, 6.59809}	{{1.82874, 2.8821}, {3., 13.6541}}	{113.764, 50.2408}	inf	38.	43.0218	{6.52481, 43.3143}	105.301	0.414752	newly	28.	52.11	{4., 65.}	163.179	0.459921	Reff	0.93521	0.534376	{0.126284, 1.20665}	57.6681	ar	411.	1756.87	{1608., 2043.}	327.462
7	R0	{1.36478, 5.96056}	{2.29042, 2.04391}	{{1.0054, 0.119191}, {3., 3.3463}}	{83.3857, 69.8008}	inf	20.	57.4437	{5.53951, 81.639}	223.232	0.402875	newly	19.	90.11	{6., 100.}	400.053	0.47666	Reff	0.848893	0.446904	{0.128525, 0.696868}	59.4464	ar	484.	1797.39	{1633., 2143.}	271.362
8	R0	{1.36335, 17.6429}	{2.84459, 6.83731}	{{2.26886, 3.78232}, {3., 9.08175}}	{110.069, 61.5853}	inf	41.	21.0071	{6.16907, 23.4495}	74.6292	0.521864	newly	19.	20.24	{5., 27.}	81.0526	0.35165	Reff	1.08932	0.593722	{0.203167, 0.79429}	59.4452	ar	484.	1587.67	{1460., 1961.}	228.031
9	R0	{1.26575, 5.28707}	{2.6921, 4.66136}	{{1.2761, 3.22375}, {3., 5.18814}}	{114.335, 23.4855}	inf	24.	32.1208	{3.61455, 63.457}	89.088	0.344937	newly	28.	48.	{4., 78.}	139.786	0.413435	Reff	0.731603	0.545256	{0.150129, 1.45587}	62.9743	ar	472.	1626.53	{1503., 1870.}	244.604
10	R0	{1.42942, 9.49905}	{2.3632, 6.61957}	{{0.18399, 3.46947}, {3., 16.7832}}	{98.4503, 60.8449}	inf	17.	74.1819	{12.3104, 110.64}	350.565	0.586092	newly	11.	56.8	{12., 84.}	426.909	0.641461	Reff	0.773316	0.335381	{0.0746277, 0.556987}	59.0923	ar	468.	1645.77	{1527., 1723.}	251.66
11	R0	{1.46827, 15.0983}	{2.62648, 4.81861}	{{1.67611, 2.9446}, {3., 7.32504}}	{78.8827, 68.1547}	inf	17.	75.8766	{3.1699, 62.0106}	374.642	0.573257	newly	6.	82.6	{2., 110.}	1293.67	0.885675	Reff	1.17902	0.458419	{0.128318, 0.976229}	68.7	ar	495.	1678.98	{1465., 2174.}	239.188
12	R0	{1.25751, 13.279}	{2.84192, 5.2027}	{{2.41219, 1.47809}, {3., 7.08167}}	{125.996, 60.8201}	inf	33.	24.3831	{4.85616, 26.1405}	67.5687	0.397365	newly	20.	26.38	{5., 36.}	88.	0.30933	Reff	1.01481	0.425974	{0.0872568, 0.691173}	72.2945	ar	366.	1620.2	{1512., 1801.}	342.678
13	R0	{1.3425, 9.16361}	{2.32565, 4.53371}	{{0.331255, 0.318314}, {3., 12.457}}	{110.521, 69.9228}	inf	14.	64.7859	{4.8583, 86.0244}	387.465	0.466059	newly	8.	53.98	{5., 100.}	589.5	0.634947	Reff	0.765225	0.438067	{0.0356247, 0.648446}	65.0775	ar	442.	1671.29	{1499., 2182.}	278.12
14	R0	{1.18936, 6.20583}	{2.51792, 6.61221}	{{1.28822, 1.74317}, {3., 13.0785}}	{111.704, 58.5901}	inf	14.	41.0628	{5.99453, 47.7216}	214.555	0.430753	newly	10.	38.37	{5., 61.}	302.5	0.472898	Reff	0.752865	0.478533	{0.192967, 0.843362}	56.3265	ar	382.	1667.82	{1545., 1914.}	336.602
15	R0	{1.27137, 9.78868}	{2.60356, 4.49383}	{{1.34529, 2.26082}, {3., 8.5406}}	{105.969, 59.5822}	inf	28.	53.8595	{9.19237, 98.121}	128.94	0.32681	newly	19.	80.08	{6., 118.}	349.158	0.441668	Reff	0.827662	0.447422	{0.143532, 0.86446}	67.7854	ar	496.	1735.33	{1564., 2257.}	249.865
16	R0	{1.23102, 13.6452}	{2.71785, 10.5729}	{{2.26536, 6.22531}, {3., 15.3802}}	{121.543, 32.1405}	inf	35.	49.9264	{8.44334, 41.65}	112.691	0.304496	newly	16.	27.16	{4., 23.}	114.375	0.296309	Reff	0.923265	0.456627	{0.125597, 0.599097}	66.5729	ar	445.	1668.7	{1514., 2122.}	274.989
17	R0	{1.27213, 13.7452}	{2.71104, 5.20365}	{{1.98852, 2.94562}, {3., 7.19702}}	{113.11, 65.5267}	inf	29.	73.574	{8.76115, 163.461}	202.12	0.367278	newly	12.	110.21	{5., 410.}	843.25	0.589806	Reff	1.02661	0.596714	{0.0466113, 1.60645}	67.4885	ar	367.	1807.41	{1549., 2647.}	392.482
18	R0	{1.25318, 11.2151}	{2.69429, 5.89455}	{{1.7346, 2.63595}, {3., 7.61493}}	{114.997, 50.4318}	inf	28.	42.7705	{2.0566, 43.695}	118.688	0.460149	newly	18.	54.56	{2., 50.}	260.	0.537103	Reff	0.917328	0.520549	{0.0324113, 0.721134}	56.3947	ar	416.	1628.6	{1471., 1906.}	291.49
19	R0	{1.46006, 8.11496}	{2.66975, 6.04611}	{{1.96166, 2.33395}, {3., 9.96766}}	{82.8519, 39.41}	inf	28.	63.2909	{2.3458, 84.9445}	179.508	0.428102	newly	24.	74.86	{2., 155.}	266.333	0.503596	Reff	0.861435	0.567256	{0.125763, 1.79445}	69.5456	ar	496.	1789.31	{1558., 2393.}	260.748
20	R0	{1.14273, 6.71261}	{2.73476, 4.02454}	{{2.33984, 1.97205}, {3., 6.64476}}	{140.284, 47.3015}	inf	18.	74.8512	{9.71938, 98.3285}	335.103	0.42811	newly	18.	86.08	{9., 125.}	395.111	0.448827	Reff	0.787341	0.473908	{0.21495, 0.836191}	51.4031	ar	362.	1780.19	{1579., 2048.}	391.765
21	R0	{1.42432, 8.23905}	{2.81863, 6.90514}	{{2.2734, 1.43702}, {3., 15.9059}}	{97.8931, 44.4855}	inf	21.	64.2647	{7.91735, 72.4066}	233.32	0.371261	newly	16.	89.69	{7., 226.}	487.688	0.454567	Reff	0.914413	0.620367	{0.185778, 1.79578}	66.9668	ar	405.	1760.37	{1561., 2404.}	334.659
22	R0	{1.29386, 17.863}	{2.48926, 3.39737}	{{1.22295, 1.11543}, {3., 5.92203}}	{94.9767, 80.981}	inf	28.	59.0906	{8.9787, 120.396}	153.3	0.322697	newly	12.	80.49	{9., 218.}	583.417	0.56602	Reff	1.06614	0.459268	{0.169781, 0.717917}	61.446	ar	488.	1678.54	{1527., 2013.}	243.963
23	R0	{1.39274, 13.0167}	{2.80326, 7.28634}	{{2.36722, 0.702811}, {3., 18.7495}}	{101.277, 59.1145}	inf	35.	42.1235	{13.9327, 66.2573}	63.3016	0.256695	newly	27.	46.83	{6., 77.}	119.37	0.36013	Reff	0.891354	0.53589	{0.198641, 0.647532}	67.9016	ar	475.	1622.02	{1465., 1772.}	241.478
24	R0	{1.32178, 15.8991}	{2.55577, 4.33364}	{{1.73832, 2.8749}, {3., 9.69857}}	{95.1578, 72.7429}	inf	48.	22.0966	{1.80688, 32.5106}	79.9477	0.666899	newly	20.	26.81	{2., 45.}	105.05	0.41095	Reff	0.896167	0.430319	{0.116287, 0.685433}	61.9879	ar	471.	1622.08	{1512., 1937.}	244.391
25	R0	{1.37568, 17.8427}	{2.76545, 8.35519}	{{2.24339, 3.80957}, {3., 11.1192}}	{101.024, 53.1731}	inf	27.	89.7412	{13.8404, 81.0487}	253.005	0.33641	newly	11.	75.94	{8., 81.}	600.909	0.496193	Reff	1.05377	0.508076	{0.169752, 0.876606}	66.137	ar	398.	1683.86	{1487., 2432.}	323.08
26	R0	{1.20993, 6.4656}	{2.55814, 11.68}	{{1.81222, 1.5674}, {3., 18.2285}}	{112.892, 131.389}	inf	9.	39.9356	{5.93628, 53.1345}	357.307	0.543685	newly	13.	42.74	{4., 70.}	273.231	0.43094	Reff	0.834852	0.555547	{0.0935586, 1.07628}	63.9951	ar	345.	1669.91	{1519., 2045.}	384.032
27	R0	{1.20548, 13.8003}	{2.69637, 3.35004}	{{1.73472, 0.230587}, {3., 8.68553}}	{123.676, 75.7249}	inf	30.	40.7024	{3.27161, 37.9165}	108.55	0.401749	newly	10.	60.17	{4., 73.}	525.9	0.525554	Reff	0.876384	0.438696	{0.197015, 0.694917}	60.2341	ar	350.	1600.79	{1474., 1707.}	357.369
28	R0	{1.3176, 14.6594}	{2.52169, 4.08104}	{{1.21422, 0.508358}, {3., 10.889}}	{95.0554, 72.161}	inf	22.	29.4152	{3.609, 32.6989}	82.0476	0.321145	newly	16.	34.96	{4., 57.}	156.5	0.418745	Reff	1.11732	0.423605	{0.162253, 0.753741}	63.1924	ar	362.	1648.4	{1554., 1790.}	355.359
29	R0	{1.28202, 6.23047}	{2.4892, 3.94101}	{{1.49402, 2.02624}, {3., 4.8704}}	{94.4663, 46.4551}	inf	11.	18.5647	{1.90526, 19.4142}	117.929	0.315055	newly	14.	30.55	{2., 21.}	179.786	0.376211	Reff	0.844851	0.40415	{0.0972773, 0.66699}	62.1196	ar	391.	1567.81	{1502., 1592.}	300.974
30	R0	{1.28177, 8.83252}	{2.65853, 5.21739}	{{2.00384, 1.61122}, {3., 10.5508}}	{107.411, 48.1725}	inf	22.	50.6049	{3.66591, 89.0943}	176.374	0.407845	newly	11.	61.7	{3., 137.}	488.	0.546894	Reff	0.848532	0.595083	{0.107109, 1.91253}	67.0945	ar	427.	1764.42	{1561., 2325.}	313.213
31	R0	{1.35886, 5.97708}	{2.66699, 8.67426}	{{2.40209, 3.55129}, {3., 12.2917}}	{98.4506, 61.2824}	inf	14.	66.8282	{9.60765, 64.2735}	390.897	0.464202	newly	13.	40.21	{8., 47.}	227.	0.387848	Reff	0.712043	0.554427	{0.217803, 0.916571}	51.5873	ar	491.	1783.24	{1609., 2196.}	263.185
32	R0	{1.27601, 17.9804}	{2.58173, 3.82874}	{{1.45266, 2.12722}, {3., 6.68404}}	{104.436, 78.7061}	inf	32.	37.883	{4.96912, 34.8842}	119.705	0.463497	newly	12.	59.28	{6., 104.}	419.333	0.431987	Reff	1.03995	0.575651	{0.125609, 1.18847}	60.3305	ar	445.	1643.32	{1491., 1908.}	269.285
33	R0	{1.3368, 11.3235}	{2.70225, 9.65912}	{{2.34754, 4.66982}, {3., 15.7066}}	{106.648, 32.8821}	inf	41.	29.6346	{7.42568, 33.7963}	62.5082	0.374499	newly	18.	21.81	{3., 35.}	81.3889	0.338221	Reff	0.838174	0.409008	{0.0492243, 0.693477}	64.0996	ar	417.	1619.01	{1530., 1791.}	288.252
34	R0	{1.21021, 11.3635}	{2.67839, 7.37241}	{{1.87661, 3.2137}, {3., 11.9739}}	{125.086, 44.0093}	inf	19.	94.3564	{11.6103, 225.58}	414.421	0.538554	newly	11.	90.84	{6., 174.}	741.455	0.633784	Reff	0.958486	0.575853	{0.176419, 1.29516}	65.8693	ar	327.	1803.74	{1567., 2496.}	451.602
35	R0	{1.53382, 10.8866}	{2.7187, 6.0189}	{{2.44226, 0.0362637}, {3., 10.4066}}	{80.1688, 44.9915}	inf	49.	55.2749	{8.60185, 73.581}	81.5545	0.33345	newly	34.	67.21	{10., 71.}	155.559	0.340906	Reff	0.963239	0.465241	{0.0681138, 0.900737}	64.3814	ar	476.	1684.09	{1532., 1788.}	253.8
36	R0	{1.20423, 6.83844}	{2.62101, 5.76007}	{{1.888, 0.244785}, {3., 7.82966}}	{117.65, 26.7162}	inf	19.	83.0279	{9.16476, 233.939}	355.774	0.511538	newly	18.	107.65	{6., 237.}	520.278	0.540798	Reff	0.714108	0.595738	{0.119504, 1.373}	62.277	ar	416.	1842.04	{1589., 2417.}	342.798
37	R0	{1.43629, 10.1548}	{2.61852, 7.8945}	{{1.98557, 4.93455}, {3., 9.17548}}	{87.0811, 32.2626}	inf	18.	58.0112	{5.92521, 123.227}	257.489	0.429945	newly	19.	39.41	{4., 70.}	161.632	0.417062	Reff	1.01977	0.396584	{0.103504, 0.763399}	64.104	ar	367.	1633.17	{1498., 1825.}	345.005
38	R0	{1.37504, 8.93428}	{2.69607, 5.0874}	{{2.00225, 2.73741}, {3., 7.02678}}	{96.0724, 43.0576}	inf	26.	35.5583	{4.25857, 50.949}	92.9764	0.353357	newly	22.	58.54	{4., 86.}	216.182	0.430397	Reff	0.764522	0.585604	{0.158871, 1.63397}	67.1116	ar	483.	1707.73	{1531., 2079.}	253.567
39	R0	{1.51637, 9.36742}	{2.38833, 9.47301}	{{0.0924921, 3.6226}, {3., 14.8168}}	{88.4353, 40.9036}	inf	18.	45.6119	{4.47602, 85.4948}	192.689	0.376656	newly	14.	38.63	{1., 84.}	233.786	0.466969	Reff	0.938633	0.482859	{0.0107088, 0.854201}	72.4076	ar	441.	1689.09	{1547., 1923.}	283.014
40	R0	{1.23658, 9.82869}	{2.78998, 8.26369}	{{2.4236, 4.82823}, {3., 14.3325}}	{125.62, 41.6795}	inf	25.	41.0794	{2.96689, 33.929}	113.292	0.30182	newly	12.	30.91	{2., 26.}	197.25	0.374532	Reff	0.746894	0.525193	{0.124584, 0.821387}	60.1993	ar	414.	1727.15	{1600., 1819.}	317.186
41	R0	{1.24247, 5.4242}	{2.41733, 7.18634}	{{0.932689, 2.25213}, {3., 13.0617}}	{105.852, 62.3367}	inf	10.	41.6787	{6.10615, 34.7747}	335.565	0.401445	newly	10.	37.08	{4., 37.}	301.	0.406073	Reff	0.861032	0.491425	{0.143525, 0.790944}	51.1602	ar	345.	1735.86	{1588., 2072.}	403.148
42	R0	{1.28208, 4.40223}	{2.78791, 7.1274}	{{2.4342, 3.60541}, {3., 8.02034}}	{117.452, 80.1509}	inf	0.1	42.0172	{4.67632, 81.3152}	41917.2	2.30761	newly	0.1	59.66	{4., 129.}	59562.	2.23842	Reff	0.847455	0.535476	{0.148576, 0.937694}	62.7832	ar	339.	1769.09	{1605., 2257.}	421.855
43	R0	{1.56773, 6.50474}	{2.60112, 6.84138}	{{1.78294, 3.49723}, {3., 12.7825}}	{70.5504, 49.9399}	inf	12.	39.9325	{3.79325, 50.7587}	255.581	0.398865	newly	11.	39.69	{3., 68.}	292.455	0.454299	Reff	0.810516	0.509622	{0.101448, 0.792311}	61.2986	ar	498.	1788.35	{1627., 2366.}	259.106
44	R0	{1.10489, 6.01941}	{2.65656, 7.93377}	{{1.306, 4.63032}, {3., 15.1678}}	{140.436, 47.1471}	inf	6.	30.8342	{3.05362, 41.6017}	428.079	0.596028	newly	6.	23.45	{3., 37.}	309.167	0.490611	Reff	0.734752	0.417322	{0.137119, 0.691483}	56.1919	ar	346.	1611.92	{1500., 1723.}	365.873
45	R0	{1.25888, 14.1926}	{2.68621, 6.15608}	{{1.79244, 3.06621}, {3., 9.55939}}	{113.381, 56.6247}	inf	40.	74.9866	{6.23777, 151.102}	137.758	0.402789	newly	16.	82.66	{3., 278.}	446.25	0.557755	Reff	0.723856	0.454318	{0.046828, 0.743117}	73.8074	ar	471.	1708.04	{1497., 2281.}	262.641
46	R0	{1.45292, 16.616}	{2.65141, 6.36888}	{{1.97756, 0.977129}, {3., 18.2123}}	{82.4887, 65.0982}	inf	41.	62.9459	{3.06517, 99.6404}	141.682	0.524594	newly	19.	81.77	{2., 182.}	385.105	0.639795	Reff	1.11003	0.599447	{0.199816, 0.938851}	64.5439	ar	494.	1745.13	{1535., 2344.}	253.265
47	R0	{1.22528, 16.6033}	{2.81133, 3.2584}	{{2.486, 0.78667}, {3., 4.88239}}	{129.444, 80.375}	inf	24.	23.2324	{2.60958, 23.2045}	59.1883	0.301567	newly	5.	47.16	{3., 49.}	859.2	0.699346	Reff	1.06599	0.491723	{0.167139, 0.879273}	60.305	ar	368.	1640.47	{1542., 1725.}	345.78
48	R0	{1.1669, 6.92715}	{2.75694, 6.21424}	{{1.95532, 4.03593}, {3., 12.4994}}	{136.262, 31.603}	inf	8.	38.4083	{7.02844, 44.0598}	390.764	0.487166	newly	4.	39.22	{4., 43.}	887.	0.677066	Reff	0.768987	0.432234	{0.0725431, 0.571215}	58.175	ar	416.	1633.28	{1505., 1835.}	292.615
49	R0	{1.32749, 16.0887}	{2.65948, 5.05125}	{{2.10959, 2.25908}, {3., 8.78073}}	{100.384, 68.6037}	inf	24.	17.8856	{5.60143, 28.1226}	46.1165	0.310901	newly	12.	21.32	{5., 41.}	107.333	0.3265	Reff	1.11376	0.467234	{0.0867026, 1.24245}	63.2352	ar	353.	1598.62	{1518., 1796.}	352.867
50	R0	{1.29636, 15.2609}	{2.87392, 4.16813}	{{2.24647, 3.34845}, {3., 4.40105}}	{121.692, 72.6875}	inf	49.	41.3596	{3.84774, 18.4573}	112.913	0.611037	newly	23.	77.87	{5., 99.}	296.565	0.403418	Reff	0.81541	0.697585	{0.0199023, 1.95788}	69.4777	ar	470.	1681.06	{1486., 2333.}	257.672
